# Extracting interface correlations from the pair distribution function of composite materials†

**DOI:** 10.1039/d1nr01922h

**Published:** 2021-07-23

**Authors:** Harry S. Geddes, Henry D. Hutchinson, Alex R. Ha, Nicholas P. Funnell, Andrew L. Goodwin

**Affiliations:** Inorganic Chemistry Laboratory, Department of Chemistry, University of Oxford South Parks Road Oxford OX1 3QR UK andrew.goodwin@chem.ox.ac.uk; ISIS Facility, Rutherford Appleton Laboratory, Harwell Science and Innovation Campus Didcot OX11 0QX UK

## Abstract

Using a non-negative matrix factorisation (NMF) approach, we show how the pair distribution function (PDF) of complex mixtures can be deconvolved into the contributions from the individual phase components and also the interface between phases. Our focus is on the model system Fe∥Fe_3_O_4_. We establish proof-of-concept using idealised PDF data generated from established theory-driven models of the Fe∥Fe_3_O_4_ interface. Using X-ray total scattering measurements for corroded Fe samples, and employing our newly-developed NMF analysis, we extract the experimental interface PDF (‘iPDF’) for this same system. We find excellent agreement between theory and experiment. The implications of our results in the broader context of interface characterisation for complex functional materials are discussed.

## Abstract 2

Using a non-negative matrix factorisation (NMF) approach, we show how the pair distribution function (PDF) of complex mixtures can be deconvolved into the contributions from the individual phase components and also the interface between phases.

## Graphical abstract

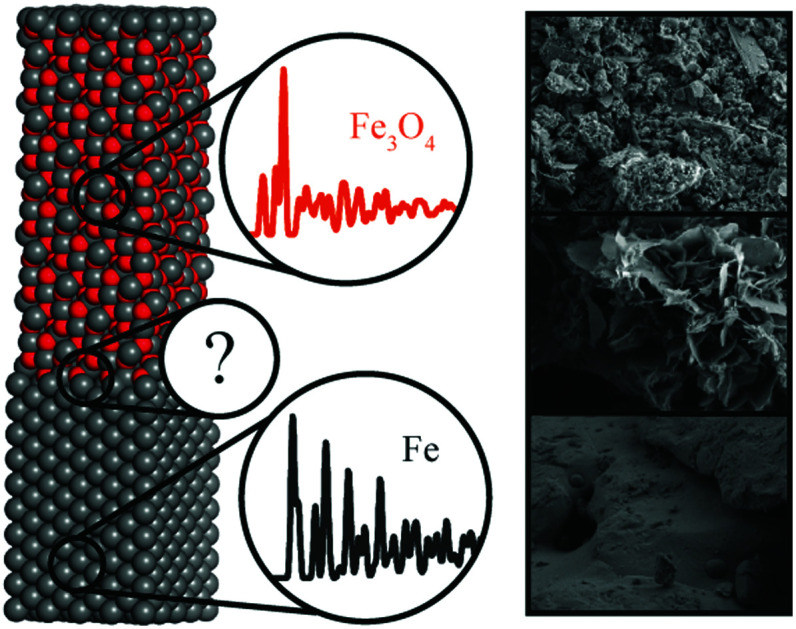

## 1. Introduction

Many important functional materials are complex mixtures that derive their properties from the interplay of various individual component phases; battery cells,^1,2^ photovoltaics,^3^ and heterogeneous catalysts^4^ are all obvious examples. In each case, the interfaces between phases are a crucial component in their own right, since they are the point at which much of the key chemistry (and/or physics) takes place.^5,6^ Hence the concept of interface engineering—*e.g.* as exploited in the LaAlO_3_∥SrTiO_3_ superconductors^7^—has emerged as a central tool in functional materials design.^3,8^

By their very nature, interfaces are notoriously more difficult to characterise than the bulk phases they connect.^9^ NMR,^10^ XAS,^11^ electron microscopy,^12^ and vibrational spectroscopy^13,14^ measurements all offer to varying degrees some experimental sensitivity to interface structure. Yet the process of translating these measurements into a picture of atomic-scale structure remains a significant general challenge.^15^ It is no surprise then that the field relies heavily on computational methods—including both *ab initio*^13^ and empirical^16,17^ approaches—to develop a collective understanding of interfaces and surface science. This is a mature science, but one that would benefit nonetheless from access to a more diverse range of experimental methods against which its results might be compared.

Here we explore the possibility that pair distribution function (PDF) measurements offer sensitivity to interface structure in a way that is strongly complementary to existing experimental and computational approaches. The PDF is determined experimentally by the inverse Fourier transform of the X-ray or neutron total scattering function—essentially a form of powder diffraction pattern that contains both Bragg and diffuse components.^18^ It represents a scattering-weighted histogram of interatomic separations. A key advantage over many other experimental techniques is that the function is sensitive at once to both short- and long-range structural correlations.^19–22^ By convention, the PDFs of complex mixtures are usually interpreted as linear sums of the individual component PDFs;^23^ such is the basis of the widely-used ‘differential PDF’ approach, for example.^24–29^ Yet this interpretation remains an approximation because it assumes the interface contribution is weak and incoherent—and so can be neglected.

**Fig. 1:**
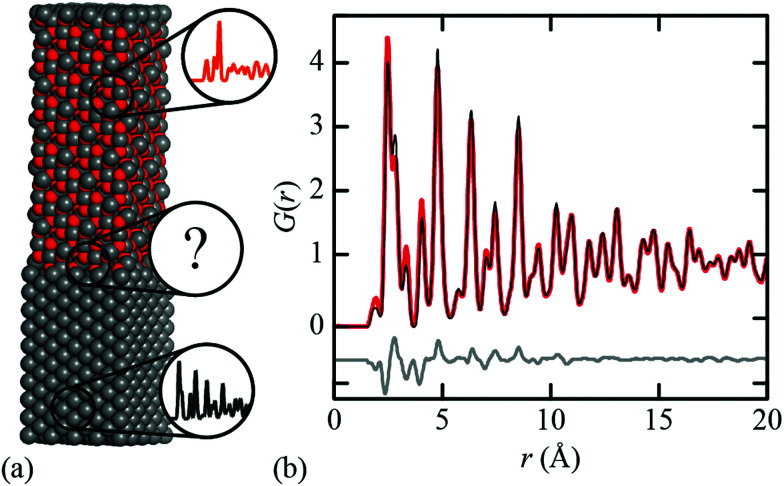
(a) Representative model used to study the Fe∥Fe_3_O_4_ interface, comprising Fe (bottom) and Fe_3_O_4_ (top); Fe atoms shown in grey, oxygen atoms in red. (b) PDFs calculated from the Fe∥Fe_3_O_4_ model (red), a two-phase fit (black) using Fe and Fe_3_O_4_ PDFs, and the corresponding difference function (grey) offset by −0.5 units.

When might this approximation break down? Consider the simple illustrative case of the Fe∥Fe_3_O_4_ interface [Fig. 1(a)] that lies at the heart of corrosion science and is industrially relevant to *e.g.* the design of pressurised heavy water reactors and industrial descaling processes.^30,31^ Whereas the conventional (Bragg) diffraction pattern of this two-phase mixture appears at face value to be a straightforward sum of the contributions from crystalline Fe and Fe_3_O_4_, its PDF cannot be accounted for entirely in terms of the Fe and Fe_3_O_4_ PDFs [Fig. 1(b)]. This is because both intra- and inter-phase correlations contribute, and the latter can be significant if a sufficient fraction of the mixture lies within an appropriate distance of the interface. For example, in a spherical particle of 30 nm diameter—typical for corrosion products^32^—nearly 35% of atoms lie within 2 nm of the surface; this fraction increases further with surface roughness. Hence composites containing nanocrystalline or amorphous phases—*e.g.* ferrihydrite^33^—are an obvious case where the interface contribution might not safely be discounted.

In this study, we show that this interface contribution to the PDF (we call it the ‘iPDF’) can actually be extracted using non-negative matrix factorisation (NMF) analysis^34^—a method only recently applied to PDF data as a means of deconvolving the function into its individual component factors.^35–37^ We begin by establishing proof-of-concept, using idealised PDF data generated from suitably-constructed models of the Fe∥Fe_3_O_4_ interface. We then report experimental X-ray total scattering measurements for controlled corrosion of polycrystalline Fe powder. We demonstrate, as anticipated, that the PDFs derived from these data contain varying contributions from three components—Fe, Fe_3_O_4_, and the Fe∥Fe_3_O_4_ iPDF. By extracting the interface PDF component and comparing against that calculated from our synthetic data, we are able to validate the interface structure model proposed on the basis of *ab initio* DFT calculations in ref. 38. Our paper concludes with a discussion of the relevance of our results to the broader study of interfaces in other families of functional materials.

## 2. Results and discussion

### Proof of concept: simulated data

By virtue of its fundamental importance to corrosion science, the Fe∥Fe_3_O_4_ interface has been heavily studied from both experimental^39–42^ and computational^38^ perspectives. The most commonly-observed interface for magnetite growth on a bcc Fe substrate is denoted crystallographically as [100](001)_Fe_∥[110](001)_Fe_3_O_4__.^39,40^ Reaction conditions are thought to affect the termination layer of the oxide component: under reducing (Fe-rich) conditions a tetrahedral Fe layer is the favoured termination layer, and in oxygen rich conditions a layer containing octahedral Fe and oxygen is preferred.^41^*Ab initio* density functional theory (DFT) calculations have shown the latter termination pattern to give the lower equilibrium interface energy.^38^

**Fig. 2:**
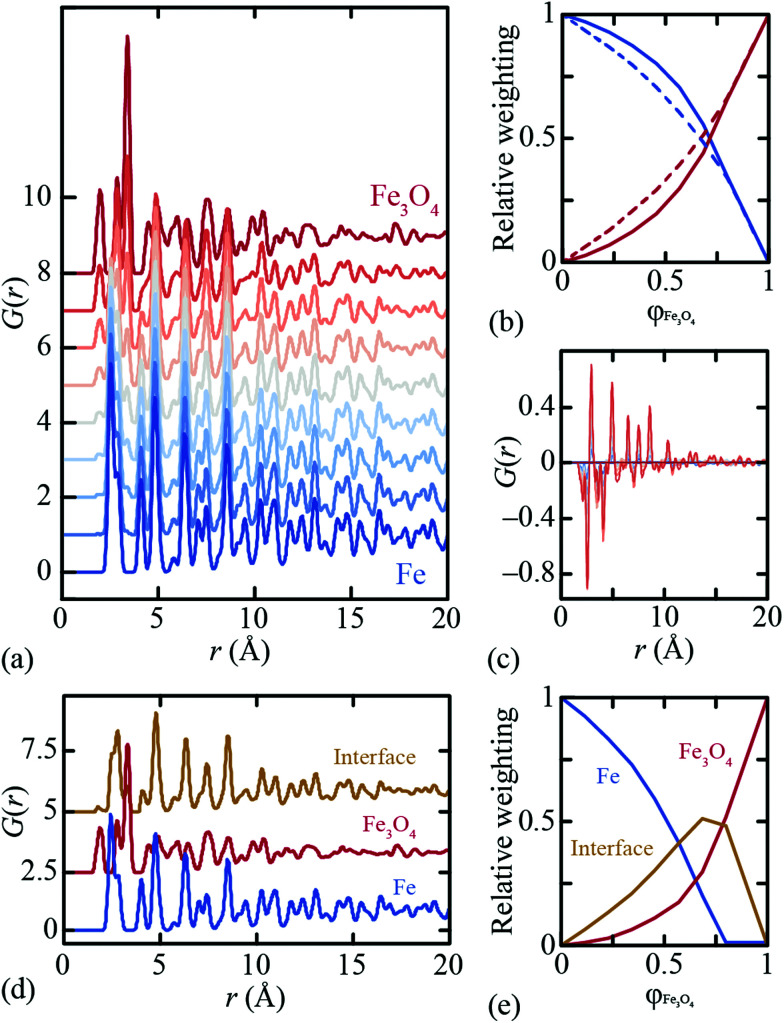
(a) PDFs calculated from Fe∥Fe_3_O_4_ models with differing ratios of each phase. (b) Relative weights (solid lines) from a two-phase fit of Fe and Fe_3_O_4_ with PDF data in (a), as a function of volume fraction of Fe_3_O_4_, φ_Fe_3_O_4__. Dashed lines indicate expected values for a system of two separate phases (*i.e.* without an interface). (c) Difference functions for these two-phase fits to the data shown in (a); blue to red colour gradient represents increasing volume fractions of Fe_3_O_4_. (d) Components (PDFs) from 3-component NMF analysis of the PDF data in (a); and (e) the relative weights from the same NMF analysis as a function of volume fraction of Fe_3_O_4_. The Fe and Fe_3_O_4_ PDFs were fixed during NMF fitting.

We used the DFT-relaxed structures of ref. 38 to build a series of atomistic models of the Fe∥Fe_3_O_4_ interface with varying Fe/Fe_3_O_4_ fractions and layer thicknesses. One step in this process is to align the in-plane lattice constants for the two phases in order to guarantee structural registry. Full details of the various models are given as ESI;† the configuration shown in Fig. 1(a) corresponds to one of these. The X-ray PDFs for all our models, together with those for pure Fe and pure Fe_3_O_4_, were calculated using PDFGui,^43^ transformed to the always-positive *G*′(*r*) normalisation of ref. 44 (we omit the prime notation hereafter for ease) and are shown in Fig. 2(a). Least squares fits to these data using linear combinations of the Fe and Fe_3_O_4_ PDFs give weighting fractions that deviate from those calculable from the known compositions [Fig. 2(b)], and also a series of difference functions with common features [Fig. 2(c)]. Our hypothesis, which we proceed to test, is that this data set can only be accounted for in terms of varying linear combinations of three PDFs: those of the two bulk-phase PDFs, and also the iPDF.

We use the Metropolis Monte Carlo NMF implementation developed in ref. 35 to deconvolve our synthetic PDF data set into its constituent components. NMF is a nonlinear fitting process that has as its variables the component PDFs and also the component weightings for each input PDF. These variables can be constrained, if so wished, in a variety of sensible ways. For example, it is possible to fix one component PDF to be that of bulk Fe, and/or another to be that of bulk Fe_3_O_4_. Likewise the component fractions can be constrained such that the bulk phase PDFs are fitted using a single NMF component. We used various combinations of constraints (including none; see ESI† for further details), and rigorously derived the results shown in Fig. 2(d and e). NMF indeed extracts three components with significant weightings, including the newly-characterised iPDF. The weighting of this component is largest for intermediate compositions; note that asymmetry of the weighting fractions in Fig. 2(e) reflects the difference in X-ray scattering powers of Fe and Fe_3_O_4_ phases. In particular, the NMF weights describe the relative contributions to the X-ray PDF at each composition, rather than *e.g.* the volume fraction of each component.

The Fe∥Fe_3_O_4_ iPDF contains features of both the Fe and Fe_3_O_4_ PDFs, but is not a linear combination of the two. This is why the iPDF fractions determined in Fig. 2(e) are much larger than the magnitude of the difference function shown in Fig. 1(b) might suggest: when fitting a composite PDF using only bulk phases, the interface contribution is approximated by a sum of the bulk-phase PDFs. By contrast, NMF allows extraction of this iPDF component in its own right. An interesting distinction between the bulk-phase and interface PDFs is that the former are independent of *r*_max_, but the latter (and its weighting) is affected by the real-space range used during the fitting process. This is sensible: the value of *r*_max_ essentially defines the interface depth to be characterised and the iPDF will be subtly different for different depths. Some further discussion on this point is given in the ESI.†

### Experimental study of Fe corrosion

**Fig. 3:**
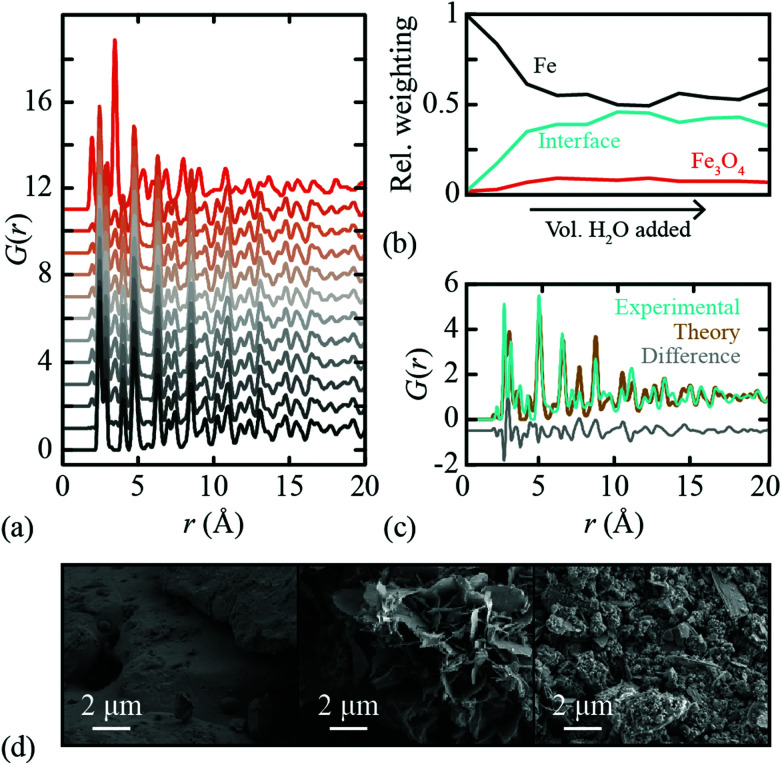
(a) X-ray PDFs of an Fe sample that has undergone controlled decomposition; grey to orange colour gradient corresponds to increasing volume of water added. The top curve is the Fe_3_O_4_ PDF for comparison. (b) Relative weights from 3-component NMF analysis of PDFs in (a). (c) Interface components from 3-component NMF analyses of model (teal) and experimental (gold) PDFs, with difference function offset vertically and shown in grey. Fe and Fe_3_O_4_ PDFs were fixed in both NMF analyses. (d) SEM images of Fe oxidation samples. The images correspond to Fe at the (left–right) start, mid-point, and end of the controlled decomposition we studied.

In order to test the efficacy of our methodology in practice, we measured X-ray PDF data for a polycrystalline Fe sample under controlled oxidation conditions. 10.0 ml of distilled H_2_O was added to 5.0 g of Fe powder in a Petri dish and left under ambient conditions to dry before being mixed. This cycle was repeated so that a total of 30.0 ml of H_2_O was added each week for a total of ten weeks, with a small fraction removed each week for PDF analysis. X-ray total scattering patterns were measured using a PANalytical Empyrean X-ray diffractometer fitted with an Ag anode (*Q*_max_ = 20 Å^−1^) and a GaliPIX3D detector. These data were processed using GudrunX^45,46^ in order to correct for background scattering, Compton scattering, multiple scattering and beam attenuation by the sample container. The resulting X-ray total scattering functions were transformed to PDFs; again we use the normalisation referred to as *G*′(*r*) in ref. 44. The corresponding X-ray PDF data are shown in Fig. 3(a), where we have included also the PDF of bulk Fe_3_O_4_ powder. Note that, despite the somewhat crude experimental conditions, there is a clear and systematic progression in measured PDFs consistent with Fe oxidation. The diffraction patterns themselves also show the evolution of a Fe_3_O_4_ component with H_2_O exposure; these data are given as ESI.† As a final check of the reproducibility of these measurements, we repeated the entire corrosion process, X-ray total scattering measurements, and subsequent data normalisation, obtaining essentially identical results (see ESI†).

Following the very same approach applied to our synthetic data set, we used Metropolis NMF to deconvolve our experimental X-ray PDF data into their constituent parts. Again, we required three NMF components to obtain satisfactory fits to data; the corresponding component fractions are given in Fig. 3(b). Note the very large interface fraction, which implies substantial interface roughness and/or nanoparticulate Fe_3_O_4_ corrosion products. Both points are borne out by scanning electron microscopy (SEM) images measured from our samples [Fig. 3(d)].

The experimental iPDF extracted from our X-ray total scattering measurements is shown in Fig. 3(c). To the best of our knowledge, this is the first experimental characterisation of the interatomic correlations at the Fe∥Fe_3_O_4_ interface. What is remarkable (in our view) is the extremely close agreement to the iPDF determined from the synthetic data set described above. Hence our data make clear that experiment is entirely consistent with theory in this case—and that the DFT model developed in ref. 38 represents a realistic picture of the interface structure in this industrially important system. In principle, measurements of the type made here might be used to examine the evolution of corrosion product growth. Our data are too coarse to draw any strong conclusions in this regard, but we note the suggestion that the interface fraction grows more rapidly than the bulk Fe_3_O_4_ phase fraction early on in the corrosion process. Crucially, these local phase fraction evolutions are related to, but distinct from, the phase fractions determined using conventional analysis of the Bragg scattering intensities.

## 3. Conclusions and future directions

We conclude that the interface PDF, or iPDF, can be extracted from experimental PDF data using the NMF-based approach outlined here—at least in the specific case we study. This iPDF can be compared against equivalent functions derived from atomistic models, hence providing an independent means of model validation. We anticipate that access to this experimental signature of interface structure will help provide important constraints on our understanding of the mechanisms of a range of functional composites, including battery materials and solid-state catalysts, and also thin-films and nanomaterials. Indeed, with the benefit of hindsight, one might in fact reinterpret the evolution of different phases determined using *e.g. operando* total scattering measurements. For example, the intermediate “Li_2_FeF_4_” phase discovered in our recent study of FeF_2_ lithiation^36^ might be equally well interpreted as the LiF∥FeF_2_ interface. No doubt there will be many other such examples.

If, as we show here, the iPDF can be calculated and measured, then in principle it can be used to refine atomistic models of interface structure. Real-space refinement packages such as PDFGui^43^ or TOPAS^47^ might straightforwardly be extended to carry out such a refinement. Doing so would bypass the need for NMF altogether: instead the PDF determined for a composite mixture might be interpreted directly in terms of crystallographic models for each component, and a description of the interface orientation. It is in this latter respect that such a refinement would differ from a conventional two-phase analysis of PDF data. There may also be cases where analysis of the interface contribution is more robust in reciprocal—rather than real—space, and we include as ESI† some discussion of the ways in which NMF might be used in this regard.

Our analysis has been understandably simplistic in many respects, and in future one might wish to explore further the role of interface shape, phase size, and even surface effects. Likewise the Fe∥Fe_3_O_4_ interface is particularly ‘clean’ in the sense that the atomic structure on either side resembles closely that of the bulk phases. It will be an interesting challenge to test measurement and interpretation of the iPDF for composites where the interface structure is very distinct over large distances; in such cases the *r*-range over which the iPDF is determined may be particularly important. Nevertheless, we hope to have demonstrated that the interface contribution to PDF data, conventionally discounted, might in fact provide enormously valuable insight into the complex structures of many functional composite materials.

## Conflicts of interest

There are no conflicts to declare.

## Supplementary Material

NR-013-D1NR01922H-s001
