# Supplementary material for: Extracting interface correlations from the pair distribution function of composite materials
Source: Nanoscale. 2021 Jul 23;13(31):13220–4. doi: 10.1039/d1nr01922h (PMC8359142; doi:10.1039/d1nr01922h)
Supplement: NR-013-D1NR01922H-s001 [file NR-013-D1NR01922H-s001.pdf]

Electronic Supplementary Material (ESI) for Nanoscale.
This journal is © The Royal Society of Chemistry 2021

Nanoscale interfaces

SUPPLEMENTARY INFORMATION

Harry S. Geddes,[a] Henry D. Hutchinson,[a] Alex R. Ha,[a] Nicholas Funnell,[b] and Andrew L. Goodwin[a*]

[a]Inorganic Chemistry Laboratory, Department of Chemistry, University of Oxford, South Parks Road, Oxford OX1 3QR, U.K.

[b]ISIS Facility, Rutherford Appleton Laboratory, Harwell Science and Innovation Campus, Didcot, OX11 0QX, U.K.

*To whom correspondence should be addressed; E-mail: andrew.goodwin@chem.ox.ac.uk

Contents

1 Experimental Methods **3**

Corrosion of Fe . . . 3

Total Scattering Measurements . . . 4

Scanning Electron Microscopy . . . 9

2 Computational Modelling of Fe||Fe_3O_4 **10**

Model Building . . . 10

PDF Calculations . . . 11

3 Bragg Diffraction **12**

Interface component in reciprocal space . . . 12

Pawley refinements of X-ray diffraction patterns . . . 13

4 Non-Negative Matrix Factorisation **15**

Simulated PDF Data . . . 15

Experimental PDF data . . . 19

Reciprocal space data from Fe||Fe_3O_4 models . . . 21

Experimental reciprocal space data . . . 23

5 References **25**

1 Experimental Methods

Corrosion of Fe

10.0 ml of distilled H_2O was added to 5.0 g of Fe powder in a Petri dish and the sample was then left under ambient conditions to dry before being mixed. This cycle was repeated so that a total of 30.0 ml of H_2O was added each week for a total of ten weeks, with a small fraction removed each week for PDF analysis. In addition a separate 5.0 g of Fe was prepared every week to ensure that a backup sample was available in case of problems with the first Fe batch over the corrosion process, but was never used. The corrosion process was repeated in an entirely separate experiment following the same experimental procedure, with the only major difference being the experiment was carried out over a shorter length of time (2 weeks compared to 10 weeks). We measured X-ray total scattering data for both controlled oxidation experiments and the results from the first experiment were reproduced closely. The iPDFs determined from the two experiments are shown in Fig. S1.

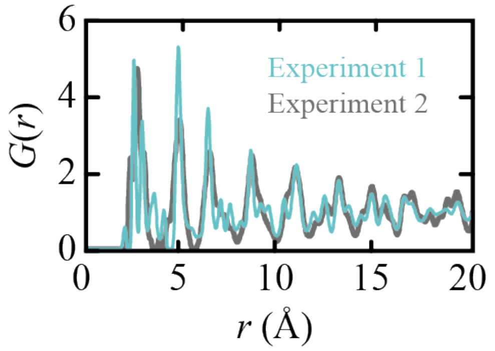

Figure S1: Comparison of the NMF-derived iPDFs two separate experimental studies of controlled decomposition of Fe. Note that the higher resolution of the iPDF from experiment 1 is because X-ray PDF data were measured using an Ag anode for experiment 1 and a Mo anode for experiment 2.

Total Scattering Measurements

X-ray total scattering patterns were measured using a PANalytical Empyrean X-ray diffractometer fitted with an Ag anode ($Q_{\text{max}} = 20$ Å^{-1}), a capillary spinner sample stage and a GaliPIX3D detector. These data were processed using GUDRUNX[S4, S5] in order to correct for background scattering, Compton scattering, multiple scattering and beam attenuation by the sample container. The resulting X-ray total scattering functions were transformed to PDFs; again we use the normalisation referred to as $G'(r)$ in Ref. S5. For comparison, samples from the second Fe corrosion experiment were measured using the same diffractometer fitted with a Mo Anode ($Q_{\text{max}} = 17$ Å^{-1}). All other instrument and data processing variables were kept as similar as possible to the first Fe corrosion.

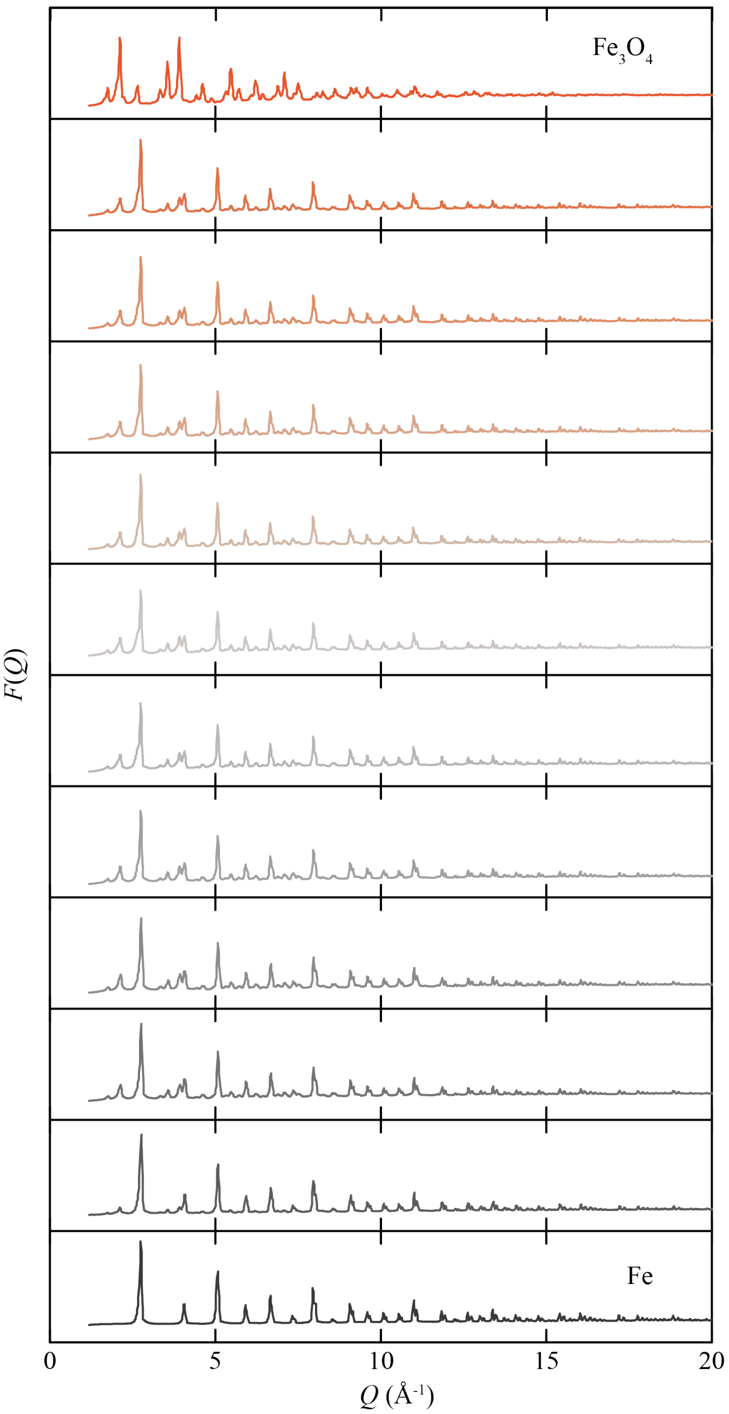

Figure S2: $F(Q)$ data from X-ray diffraction measurements from the first controlled decomposition of Fe. Grey to orange colour gradient corresponds to increasing volume of water added.

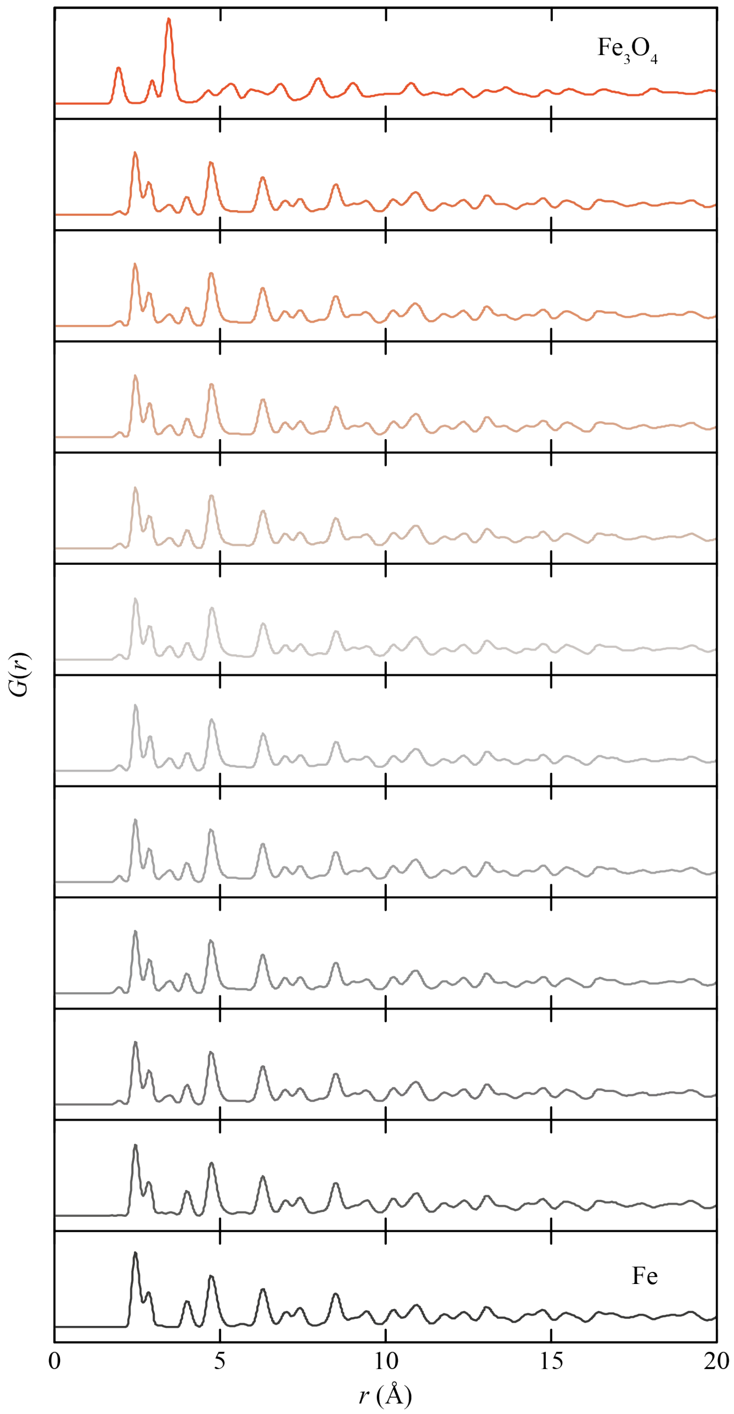

Figure S3: X-ray PDFs from the first controlled decomposition of Fe; grey to orange colour gradient corresponds to increasing volume of water added. The same data are presented in Fig. 3(a) of the main text.

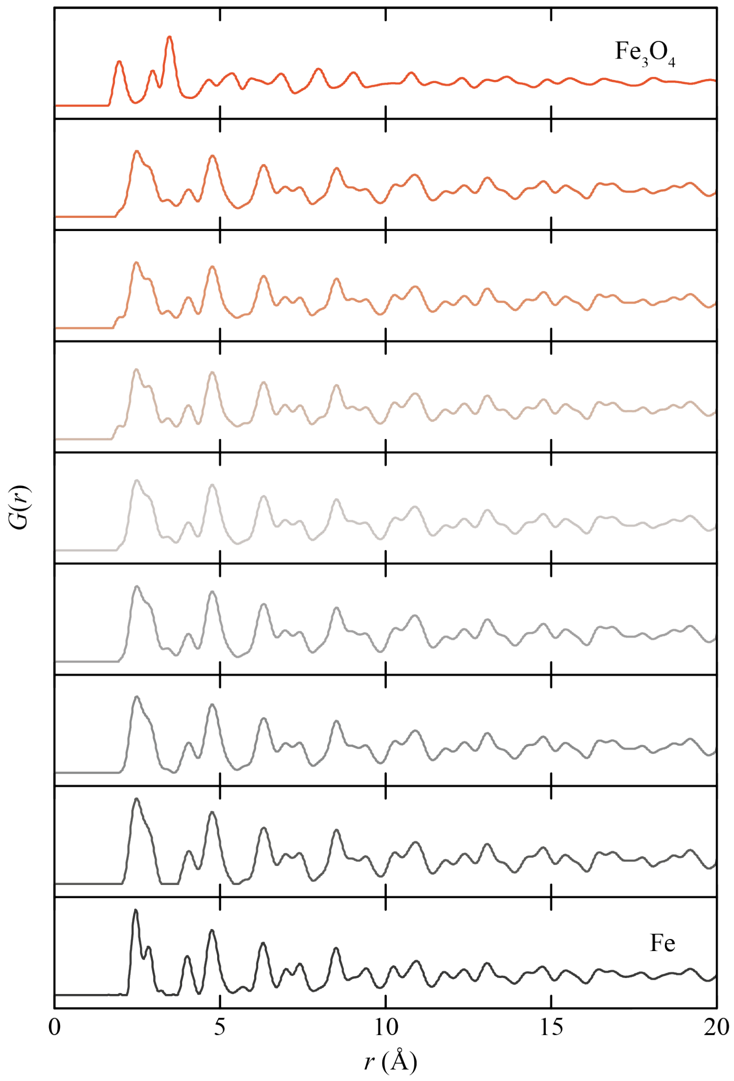

Figure S4: X-ray PDFs from the second controlled decomposition of Fe; grey to orange colour gradient corresponds to increasing volume of water added. Note the lower resolution here is because a Mo anode ($Q_{\text{max}} = 17$ Å^{-1}) was used compared to an Ag anode in experiment 1.

In order to determine which iron oxide phases were present in partially-decomposed Fe, the PDF of the sample was compared with those measured from pure Fe_3O_4, Fe_2O_3 and $Fe(OH)_2$ are in Fig. S5. The presence of Fe_3O_4 is evidenced by the developing peaks at 2.9 Å and 3.5 Å in the PDF of decomposed Fe. These peaks correspond to Fe–O distances in Fe_3O_4 and the increasing intensities of these two peaks indicate the growth of Fe_3O_4 in the Fe sample.

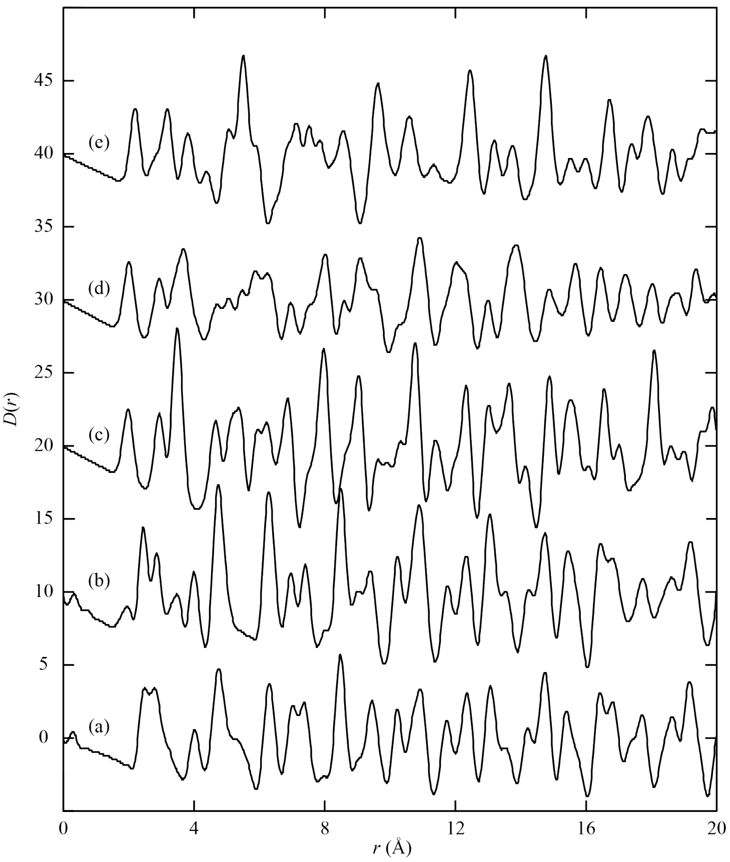

Figure S5: X-ray PDFs of (a) Fe powder, (b) Fe that has undergone controlled decomposition, (c) Fe_3O_4, (d) α-Fe_2O_3, and (e) $Fe(OH)_2$.

Scanning Electron Microscopy

Powdered samples of Fe were scattered onto Cu tape affixed onto aluminium 12.5 mm stubs. Carbon was evaporated onto the surface of the prepared stubs to a thickness of 7.5 nm; this is to suppress charging caused by the electron beam. Samples were observed using a Zeiss Merlin scanning electron microscope, at an accelerating voltage of 3 kV and probe current 100 pA. energy-dispersive X-ray analysis was carried out under the same conditions, using an Oxford instruments X-max 80 mm detector and Aztec software version 3.0.

2 Computational Modelling of $Fe||Fe_3O_4$

Model Building

To study the $Fe||Fe_3O_4$ interface we built atomistic models with varying Fe/Fe_3O_4 fractions and layer thicknesses. We used the DFT-relaxed structures of Ref. S1 and the Fe_3O_4 unit cell length in the $[001]$ direction was reduced (from 8.396 Å to 8.108 Å) in order to align with the Fe lattice parameter and guarantee structural registry. We also built atomistic models by aligning the cell dimensions the other way (*i.e.* stretching the Fe unit cell to fit Fe_3O_4). The iPDF from NMF of X-ray PDFs from these models agree less well with the experimental iPDF. The models described in the main text are outlined in Table S1; the volumes of the various models are different because the layer thickness of Fe and Fe_3O_4 are different.

Figure S6 shows the structure at the $Fe||Fe_3O_4$ interface in the atomistic models. Figure S6(a) and (b) show the unit cells of Fe and Fe_3O_4 respectively with the blue planes indicating the orientation of the plane of the interface in the model shown in Figure S6(c).

a (Å)	b (Å)	c (Å)	Volume (Å^3)	c_{Fe} (Å)	$c_{Fe_3O_4}$ (Å)	φ_{Fe}	$\varphi_{Fe_3O_4}$
8.108	8.108	200.663	13191.501	200.663	0	1	0.0
8.108	8.108	202.881	13337.308	174.863	26.661	0.88	0.11
8.108	8.108	202.616	13319.902	151.930	49.295	0.76	0.23
8.108	8.108	202.351	13302.495	128.997	71.949	0.65	0.34
8.108	8.108	202.086	13285.091	106.065	94.610	0.54	0.45
8.108	8.108	201.822	13267.685	83.132	117.274	0.43	0.57
8.108	8.108	201.557	13250.279	60.199	139.939	0.31	0.68
8.108	8.108	201.292	13232.873	37.266	162.605	0.20	0.80
8.108	8.108	206.496	13574.964	0	206.496	0	1

Table S1: Details of atomistic models used to study the $Fe||Fe_3O_4$ interface.

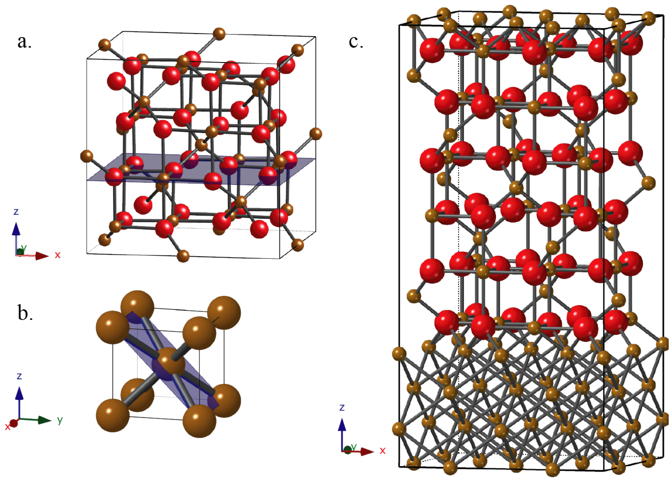

Figure S6: Unit cells of (a) Fe, (b) Fe_3O_4 and (c) an example cell used to model the Fe$||$$Fe_3O_4$ interface. Blue planes in (a) and (b) indicate orientation of the interface in the model in (c).

PDF Calculations

X-ray PDFs were calculated for the series of atomistic models of the Fe$||$$Fe_3O_4$, including Fe and Fe_3O_4 using the PDFgui software.[S2] When calculating PDFs the parameters were set to mirror the conditions of the experimental measurements as closely as possible. Values of $Q_{\mathrm{max}} = 20\,\text{Å}^{-1}$ and $Q_{\mathrm{damp}} = 0.06\,\text{Å}^{-1}$ were used, and isotropic atomic displacement parameters for all atoms were set as 0.01 Å^2.

3 Bragg Diffraction

Interface component in reciprocal space

In the main text we discuss how the conventional (Bragg) diffraction pattern of a two-phase mixture of Fe and Fe_3O_4 appears to be essentially a straightforward sum of the contributions from crystalline Fe and Fe_3O_4. To illustrate this point we calculated the diffraction pattern from an Fe||Fe_3O_4 interface model—a model with $\varphi_{\mathrm{Fe_3O_4}} = 0.45$ was used (Table S1). We find a diffraction pattern that contains peaks characteristic of both crystalline Fe and Fe_3O_4—the diffraction patterns are given in Fig S7. Note that this component does in fact also contain a weak diffuse component, and the Bragglike reflections are somewhat broadened with respect to the traces for the individual bulk phases.

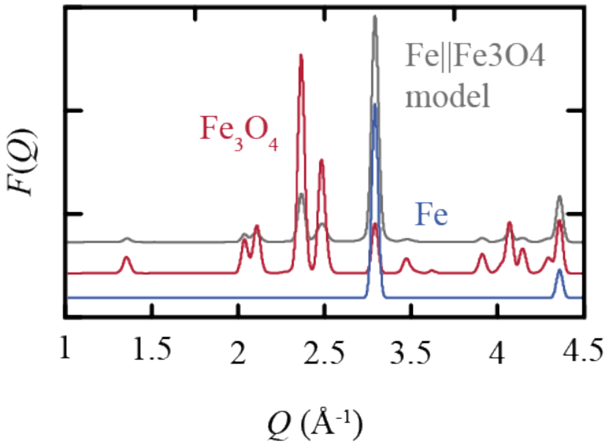

Figure S7: Calculated Bragg Diffraction patterns for Fe (blue), Fe_3O_4 (red) and an Fe||Fe_3O_4 interface model (grey).

Pawley refinements of X-ray diffraction patterns

Pawley refinements were carried out using TOPAS Academic (version 4.1).[S3] The measured scattering patterns of corroded Fe samples were fitted with the diffraction patterns of Fe and Fe_3O_4 present in the sample using Pawley refinement. Scale factors and the crystallite size were also refined. A representative Pawley refinement is illustrated in large format in Fig. S8, and the corresponding fits for all the room-temperature powder diffraction data for the corroded Fe samples are shown in Fig. S9. The key point here is that the experimental reciprocal space data can be reasonably well accounted for in terms of a conventional two-phase refinement, in spite of the fact that we know the interface contribution to be significant.

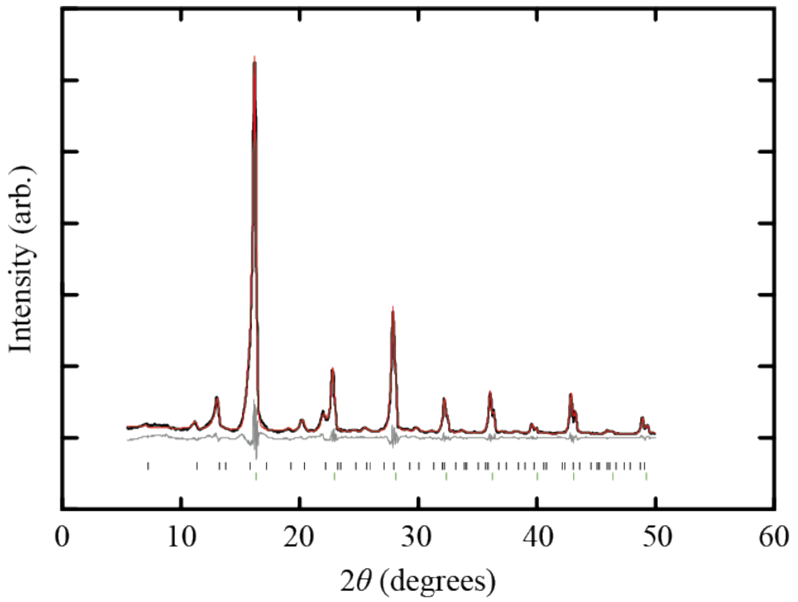

Figure S8: Pawley fit of the X-ray diffraction pattern for the first sample of corroded Fe. X-ray diffraction pattern (black), Pawley refinement (red), difference function (grey) and the symmetry-allowed reflections for Fe (green tick marks) and Fe_3O_4 (black tick marks).

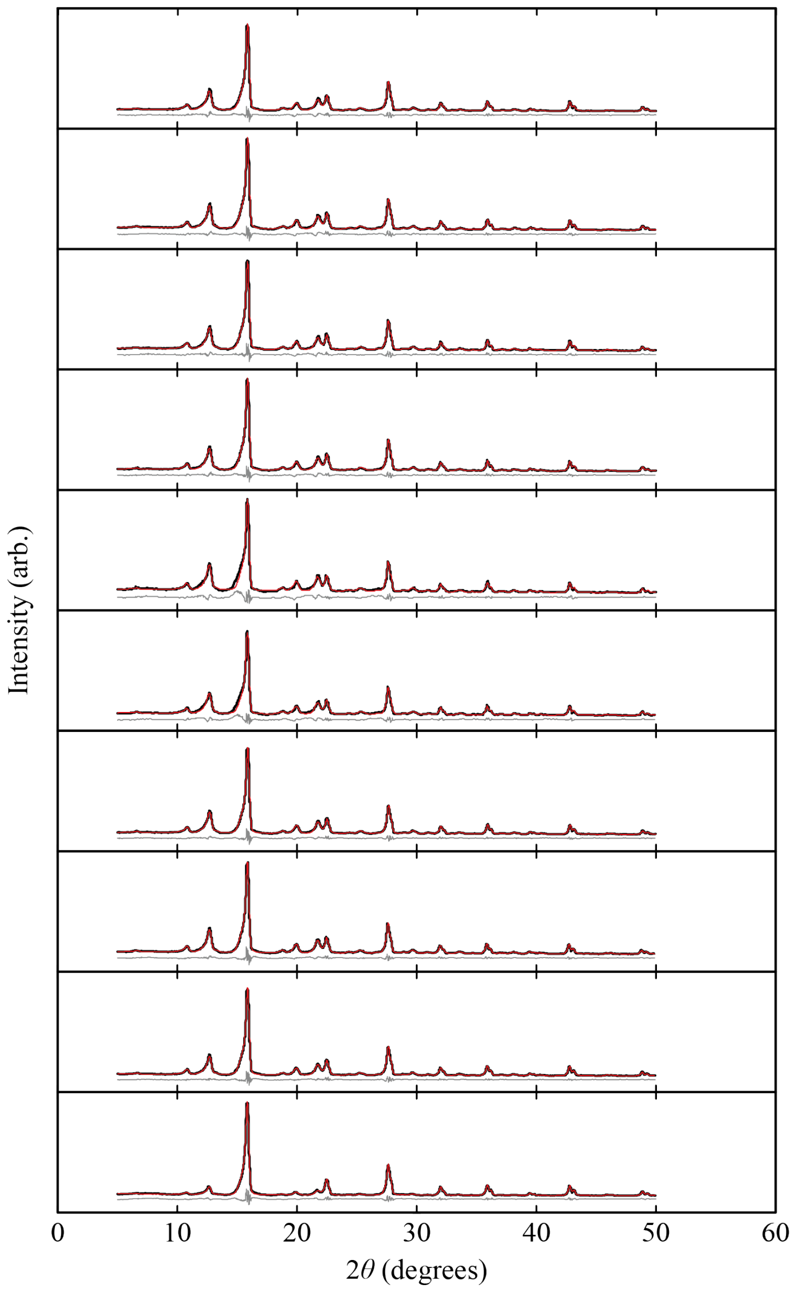

Figure S9: X-ray diffraction patterns (black lines), Pawley refinements (red lines) and difference functions (grey lines) for the corroded Fe samples at room temperature. The lowest panel is the least corroded samples, and the top is the more corroded sample.

4 Non-Negative Matrix Factorisation

We use the Metropolis Monte Carlo NMF implementation developed in Ref. S6 to deconvolve PDF data into its constituent components. NMF is a nonlinear fitting process that has as its variables the component PDFs and also the component weightings for each input PDF. These variables can be constrained, if so wished, in a variety of sensible ways. For example, it is possible to fix one component PDF to be that of bulk Fe, and/or another to be that of bulk Fe_3O_4. Likewise the component fractions can be constrained such that the bulk phase PDFs are fitted using a single NMF component.

In this study, the task for NMF was to identify the three fundamental components $G_i^*(r)$ ($i = 1, 2, 3$) and weights w_{ij} ($j = 1, 2, \ldots, 9$ for synthetic data; $j = 1, 2, \ldots, 12$ for experimental PDF data) so as to minimise $|\mathbf{G}^{\rm calc}(r) - \mathbf{G}^{\rm exp}(r)|^2$, where $G_j^{\rm calc}(r) = \sum_{i=1}^{3} w_{ij} G_i^*(r)$ are the elements of $\mathbf{G}^{\rm calc}(r)$. We applied the additional constraints that $G_i^*(r)$ is positive for all i and r, and that $\sum_{i=1}^{3} w_{ij} G_i^*(r) = 1$ for all j.

Simulated PDF Data

In the main text we use NMF to deconvolve our synthetic PDF data into 2 components, and into 3 components; and for both we fixed the PDFs of Fe and Fe_3O_4. In each case the relative weightings were determined from random initial values, and for the 3-component NMF the iPDF was derived. NMF fits for the 2- and 3-component NMF analyses are shown in Fig. S10 and Fig. S11 respectively. If either the Fe or Fe_3O_4 PDFs are not fixed (*i.e.* given random initial values and allowed to vary) then NMF fails to distinguish the iPDF from the pure phases.

To help understand how the value of $r_{\rm max}$ affects the iPDF, 3-component NMF analyses of the synthetic PDF data was repeated using the same method as presented in the main text, but with $r_{\rm max}$ values of 10 Å and 7 Å—the results are presented in Fig. S12 and Fig S13 respectively. For smaller $r_{\rm max}$ values the NMF weight of the interface component increases as the iPDF captures fewer correlations that exist in bulk Fe or Fe_3O_4.

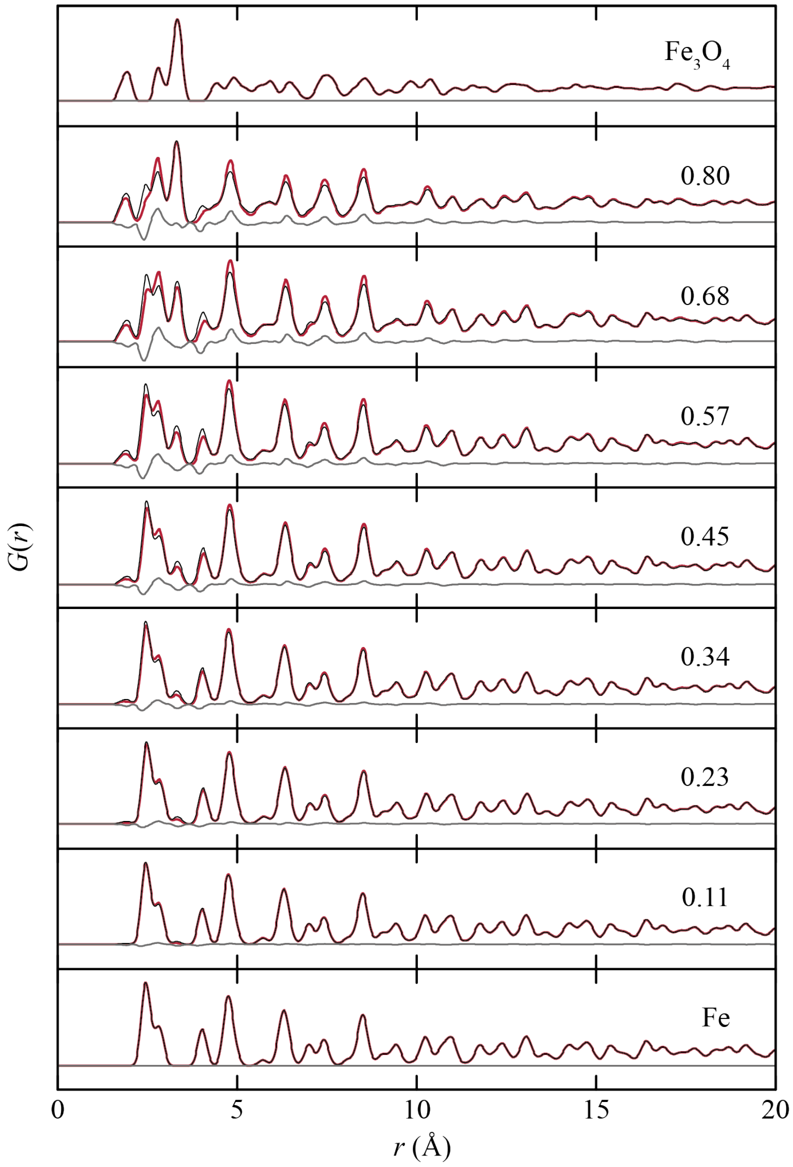

Figure S10: Synthetic X-ray PDF data calculated from Fe||Fe_3O_4 model (red), with NMF fits (black) from a 2-component NMF, and difference functions between the PDF data and NMF reconstructed data (grey). Numbers for each plot is the volume fraction of Fe_3O_4 for each model.

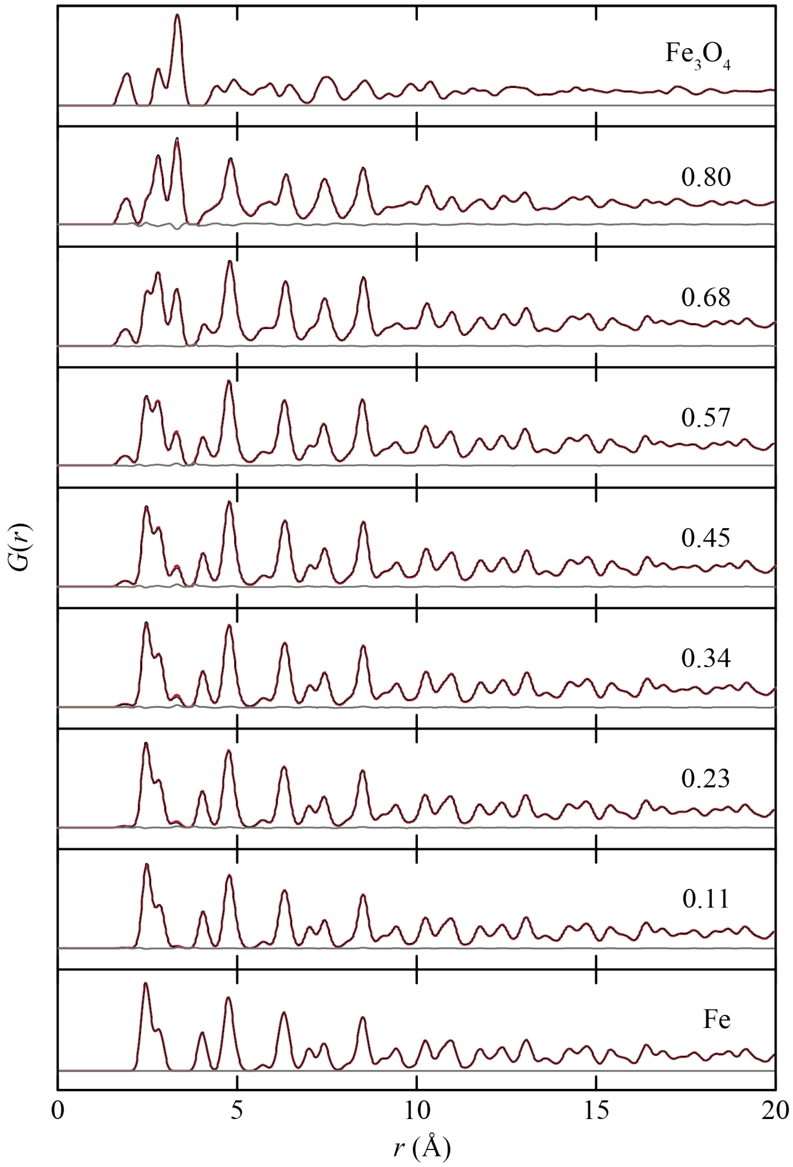

Figure S11: Synthetic X-ray PDF data calculated from Fe||Fe_3O_4 model (red), with NMF fits (black) from a 3-component NMF, and difference functions between the PDF data and NMF reconstructed data (grey). Numbers for each plot is the volume fraction of Fe_3O_4 for each model.

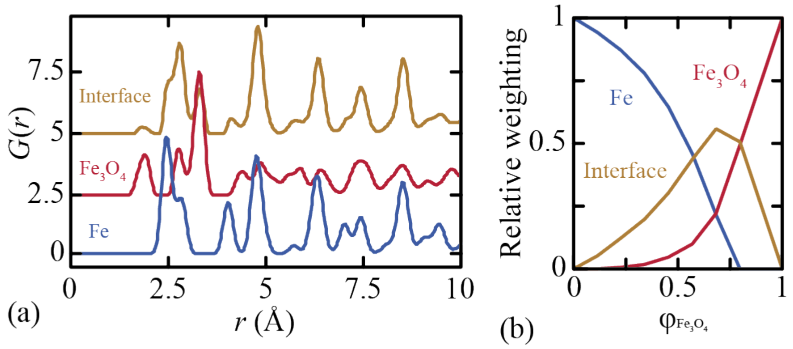

Figure S12: NMF with $r_{\mathrm{max}} = 10\,\text{Å}$ (a) Components (PDFs) from 3-component NMF analysis of PDF data from Fe||Fe_3O_4 model, and (b) the relative weights from the same NMF analysis as a function of volume fraction of Fe_3O_4. Fe and Fe_3O_4 were fixed during NMF.

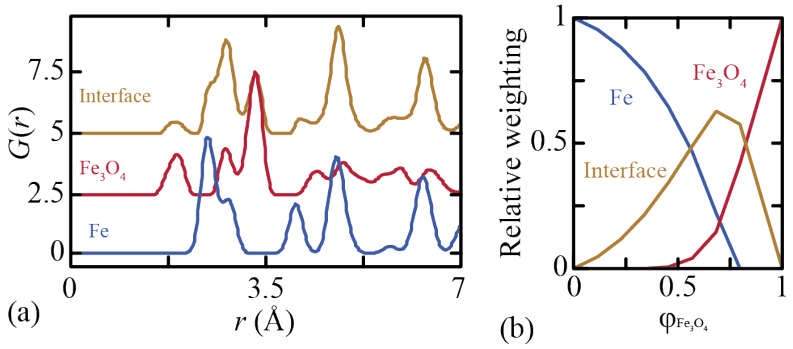

Figure S13: NMF with $r_{\mathrm{max}} = 7\,\text{Å}$ (a) Components (PDFs) from 3-component NMF analysis of PDF data from Fe||Fe_3O_4 model, and (b) the relative weights from the same NMF analysis as a function of volume fraction of Fe_3O_4. Fe and Fe_3O_4 were fixed during NMF.

Experimental PDF data

In the main text we use NMF to deconvolve experimental X-ray PDF data of Fe that has undergone controlled decomposition into 3 components; the PDFs of Fe and Fe_3O_4 were fixed during the NMF. The NMF fits for the results presented in the main text are shown in Fig S14. To test our Metropolis Monte Carlo NMF implementation, we used a similar approach to the one described in the main text but the PDFs of Fe and Fe_3O_4 were not fixed. The results of NMF with only the PDF of Fe fixed (*i.e.* Fe_3O_4 PDF allowed to vary from random initial values), and with no components fixed are given in Fig S15 and Fig S16 respectively. In both cases the iPDFs derived have similar features to the experimental iPDF from the main text with slight changes in relative peak intensities. There are also small changes in the NMF weights for the three different NMF analyses.

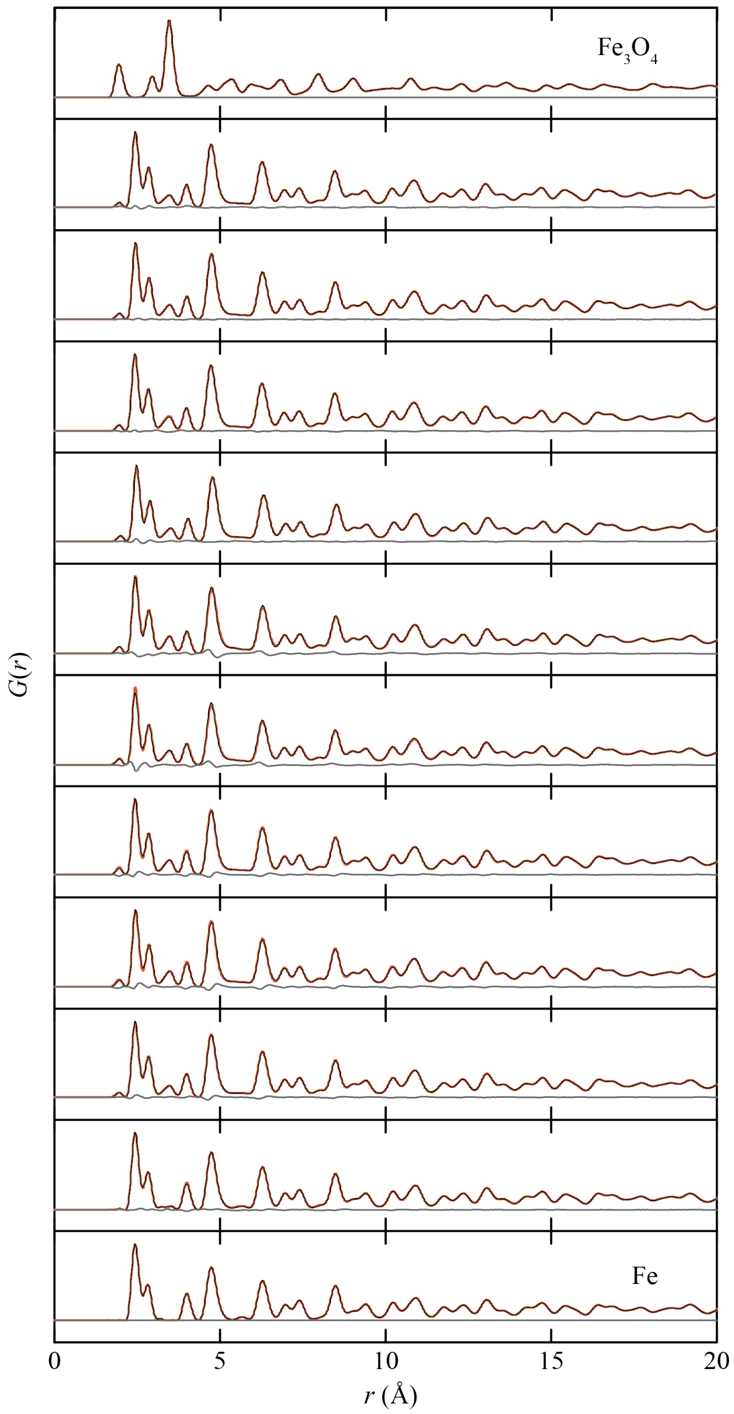

Figure S14: X-ray PDFs of Fe that has undergone controlled decomposition (red), with NMF fits (black) from a 3-component NMF, and difference functions between the PDF data and NMF reconstructed data (grey). Bottom to top represents increased corrosion.

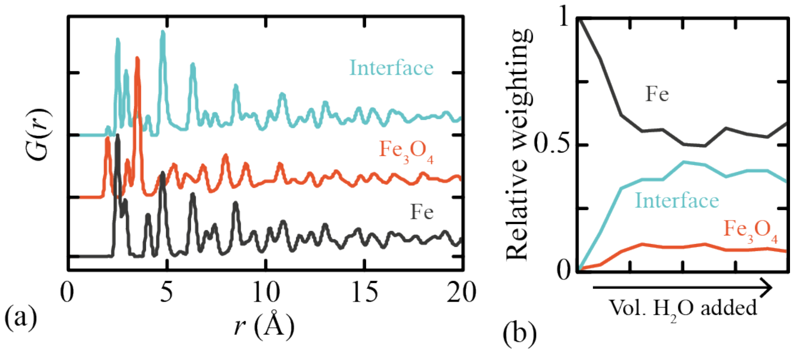

Figure S15: (a) Components (PDFs) from 3-component NMF analysis of X-ray PDF data of Fe that has undergone controlled decomposition. (b) the relative weights from the same NMF analysis. Only Fe was fixed during NMF; Fe_3O_4 and the iPDF were derived by NMF.

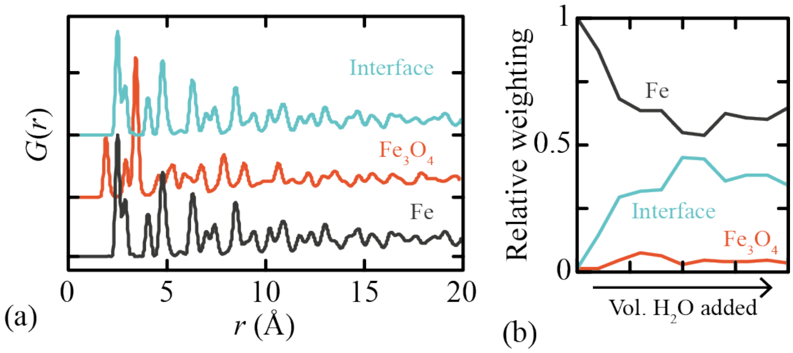

Figure S16: (a) Components (PDFs) from 3-component NMF analysis of X-ray PDF data of Fe that has undergone controlled decomposition. (b) the relative weights from the same NMF analysis. None of the components (PDFs) were fixed during NMF.

Reciprocal space data from Fe$||$$Fe_3O_4$ models

While our focus is on the interpretation of X-ray total scattering data in terms of its real-space transforms, we include for completeness an additional NMF analysis here that uses the reciprocal space data. Our starting point was to calculate the total scattering function for the DFT-driven synthetic models. We used a variant of the total scattering function which we call $F'(Q)$ chosen so that it is (a) positive everywhere, (b) satisfies $F'(Q \to 0) = 0$, (c) satisfies

$F'(Q \to \infty) = 1$, and (d) is normalised such that $\int_0^{Q_{\max}} F'(Q)\,\mathrm{d}Q$ is constant. The corresponding data are shown in Fig. S17, and the corresponding three-phase NMF results are given in Fig. S18. Note the similarity between panel (c) of that figure and Fig. 2(c) of the main text.

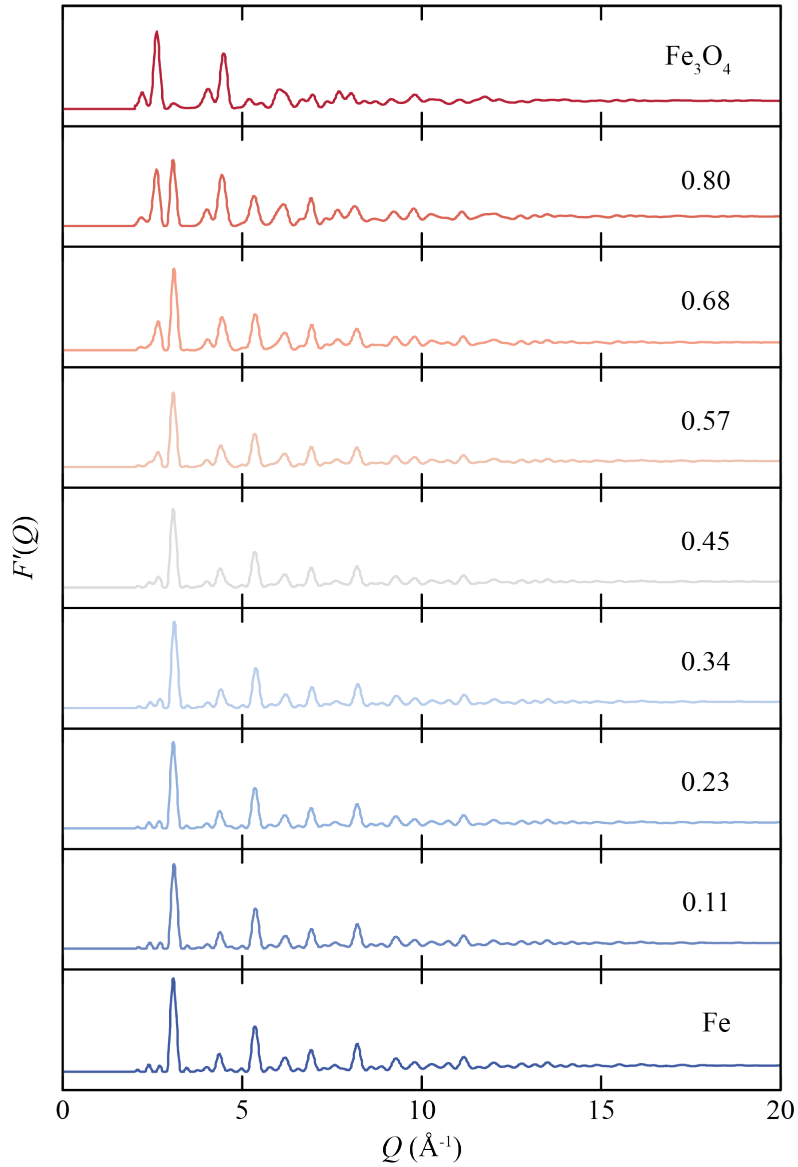

Figure S17: F(Q) data from Fourier transform of X-ray PDF data calculated from Fe||Fe_3O_4 models. Numbers for each plot is the volume fraction of Fe_3O_4 for each model.

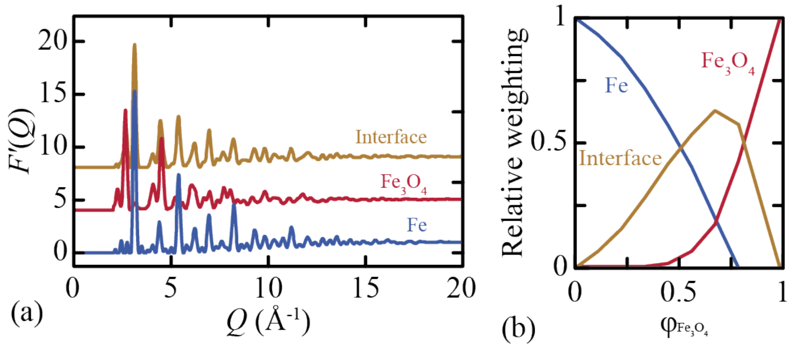

Figure S18: (a) Components, $F(Q)$, from 3-component NMF analysis of XRD data calculated from Fe||Fe_3O_4 models. Fe_3O_4 and the NMF-derived interface component are each shifted vertically by 4 units. (b) the relative weights from the same NMF analysis. Fe and Fe_3O_4 were fixed during NMF.

Experimental reciprocal space data

We used the same approach to carry out a three-component NMF refinement of the experimental total scattering data, normalised in the same way as described above. The corresponding interface $F'(Q)$ function contains a mixture of Bragglike and diffuse features [Fig. S19(a)], as expected, and the evolution of different phase components tracks oxidation in qualitatively the same way as determined using the real-space transforms: *c*f Fig. S19(b) and Fig. 3(b) of the main text. Note that we do not expect quantitative agreement here in phase fractions, since the meaning of the iPDF is linked to the real-space range over which the NMF analysis is carried out.

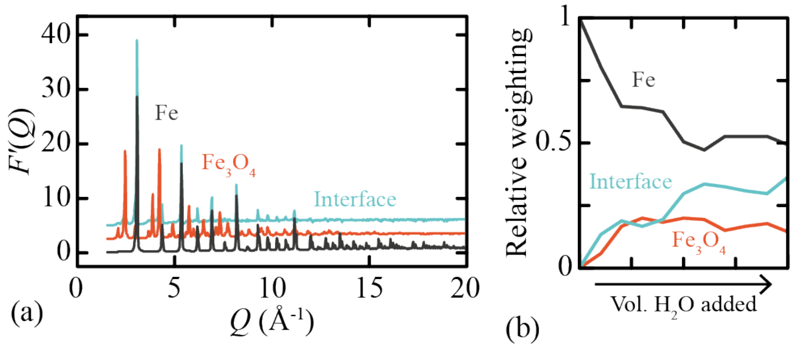

Figure S19: (a) Components, $F(Q)$, from 3-component NMF analysis of XRD data of Fe that has undergone controlled decomposition. Fe_3O_4 and the NMF-derived component are each shifted vertically by 5 units. (b) the relative weights from the same NMF analysis. Fe and Fe_3O_4 were fixed during NMF.

5 References

(S1) Forti, M. D.; Alonso, P. R.; Gargano, P. H.; Balbuena, P. B.; Rubiolo, G. H., *Surf. Sci.*, **2016**, *647*, 55–65.

(S2) Farrow, C. L.; Juhas, P.; Liu, J. W.; Bryndin, D.; Božin, E. S.; Bloch, J.; Proffen, T.; Billinge, S. J. L., *J. Phys.: Condens. Matter*, **2007**, *19*, 335219.

(S3) A. A. Coelho, *TOPAS-Academic, version 4.1 (computer software)*, Coelho Software, Brisbane.

(S4) Soper, A. K.; Barney, E. R. *J. Appl. Crystallogr.*, **2011**, 714–726.

(S5) A. K. Soper, *GudrunN and GudrunX: Programs for Correcting Raw Neutron and X-ray Diffraction Data to Differential Scattering Cross Section Tech. Rep. RAL-TR-2011-013* (Rutherford Appleton Laboratory, 2011).

(S6) Keen, D. A. *J. Appl. Cryst.* **2001**, 34, 172–177.

(S7) Geddes, H. S.; Blade, H.; McCabe, J. F.; Hughes, L. P.; Goodwin, A. L., *Chem. Commun.*, **2019**, *55*, 13317–13474.
